# What are task-sets: a single, integrated representation or a collection of multiple control representations?

**DOI:** 10.3389/fnhum.2013.00524

**Published:** 2013-09-03

**Authors:** Dragan Rangelov, Thomas Töllner, Hermann J. Müller, Michael Zehetleitner

**Affiliations:** ^1^Department Psychologie, Allgemeine und Experimentelle Psychologie I, Ludwig-Maximilians-Universität MünchenMünchen, Germany; ^2^Birkbeck College, University of LondonLondon, UK

**Keywords:** task switching, task sets, attentional selection, perceptual processing, electroencephalography, executive control

## Abstract

Performing two randomly alternating tasks typically results in higher reaction times (RTs) following a task switch, relative to a task repetition. These task switch costs (TSC) reflect processes of switching between control settings for different tasks. The present study investigated whether task sets operate as a single, integrated representation or as an agglomeration of relatively independent components. In a cued task switch paradigm, target detection (present/absent) and discrimination (blue/green/right-/left-tilted) tasks alternated randomly across trials. The target was either a color or an orientation singleton among homogeneous distractors. Across two trials, the task and target-defining dimension repeated or changed randomly. For task switch trials, agglomerated task sets predict a difference between dimension changes and repetitions: joint task and dimension switches require full task set reconfiguration, while dimension repetitions permit re-using some control settings from the previous trial. By contrast, integrated task sets always require full switches, predicting dimension repetition effects (DREs) to be absent across task switches. RT analyses showed significant DREs across task switches as well as repetitions supporting the notion of agglomerated task sets. Additionally, two event-related potentials (ERP) were analyzed: the Posterior-Contralateral-Negativity (PCN) indexing spatial selection dynamics, and the Sustained-Posterior-Contralateral-Negativity (SPCN) indexing post-selective perceptual/semantic analysis. Significant DREs across task switches were observed for both the PCN and SPCN components. Together, DREs across task switches for RTs and two functionally distinct ERP components suggest that re-using control settings across different tasks is possible. The results thus support the “agglomerated-task-set” hypothesis, and are inconsistent with “integrated task sets.”

## Introduction

Surviving in an environment in which both internal and external conditions change dynamically presupposes an ability to change between control settings for old and new tasks. A successful switch implies that the set of expectations about the environment (the topic of the present special issue) which was relevant in the previous task episode is replaced by one appropriate for the task at hand. Such switching processes are usually investigated in paradigms in which two or more different tasks vary across trials, requiring a change, on task-switch trials, in the internal control settings so as to fit the current task requirements. In *cued task switching*, prior to the stimulus display's onset, a cue is presented specifying the task to be performed on the upcoming trial. Across two consecutive trials, the task can either repeat or change. Reaction times (RTs) and errors are typically elevated for task switches relative to repetitions (Allport et al., 1994; Rogers and Monsell, 1995). Such task switch costs (TSCs) imply the existence of extra, time-consuming processes invoked on task switch trials, but not (or to a lesser degree) on repetition trials. To comprehensively account for TSCs, answers to two related, yet separable questions are necessary: first, what cognitive mechanisms give rise to the TSCs, and, second, how are the representations on which these mechanisms operate organized? The present study focused on the latter issue—more precisely, on whether or not having to change *some* expectations automatically triggers a change in *all* expectations about task-relevant properties of the environment.

### Determinants of TSCs

The available literature offers two dominant approaches to the question of what cognitive *mechanisms* give rise to TSCs. According to the first, TSCs reflect the extra time it takes to reconfigure control settings from the previously relevant to the currently relevant task demands (Monsell and Driver, 2000; Monsell et al., 2003). Reconfiguration is achieved by means of a special executive function (or set of functions) which is active on task switches and inactive on task repetitions. An alternative approach assumes that TSCs reflect interference between the previously relevant and the currently required control settings, which are concurrently active on task switch trials (Allport et al., 1994; Gilbert and Shallice, 2002; Waszak et al., 2003). TSCs arise because the interference is weaker on task repetition than on task switch trials. Finally, a hybrid, reconfiguration-interference account has also been proposed, postulating that TSCs reflect a mixture of reconfiguration and interference processes (Meiran, 1996, 2000, but see Meiran et al., 2008). Critically, irrespectively of what mechanisms produce TSCs, all accounts assume that performance of a task is controlled by a set of representations that, following a task switch, are no longer appropriate. Thus, to meet the changed environmental demands, the control representations would have to change, too. Given this, the present study was designed to address the question of what *representations* change across task switches.

Conceptually, tasks can differ in all or some of the following respects: (i) criteria for spatial-attentional selection of the task-relevant stimulus; (ii) criteria for the identification of task-relevant stimulus properties; and (iii) task-appropriate stimulus-response (S-R) mappings. The set of cognitive representations specifying these criteria is considered collectively to constitute the task set. The available literature supports the notion of composite task set representations. For instance, Meiran (2000) demonstrated that while switching identification criteria can be performed in advance, actually performing the task is necessary for switching S-R mappings. Furthermore, Hübner et al. (2001) showed that the magnitude of TSCs increases with the number of task set components to be switched. Finally, a number of electrophysiological and imaging studies revealed neural correlates of a switch to co-vary with what task set component is being switched (Rushworth et al., 2002, 2005; Ravizza and Carter, 2008; Chiu and Yantis, 2009; Esterman et al., 2009; Hakun and Ravizza, 2012). In summary, the available evidence converges on the view that task sets consist of several dissociable representations controlling different cognitive processes in the stimulus-response chain. However, it remains unclear whether, on task switch trials, different components are changed relatively independently of each other, as would be predicted by the notion of “agglomerated task sets.”

Studies investigating whether or not it is possible to change only those task set components that require a change and to reuse shared components across different tasks yielded somewhat inconsistent findings. For example, Arrington et al. (2003) asked their participants to report, on different trials, either the stimulus height, width, color, or luminance. Smaller TSCs were found for switches across similar tasks (e.g., switching from color to luminance discrimination) relative to switches across dissimilar tasks (e.g., from height to luminance discrimination), suggesting that some reusing of control representations across different tasks is possible. By contrast, Vandierendonck et al. (2008) had their participants discriminate either the parity (odd/even) or the magnitude (greater/smaller than four) of stimuli consisting of several identical digits (e.g., five instances of the digit three). On different trials, either the number of digits or the digits themselves were task-relevant. The critical comparison was between trials on which both the identification criterion (parity vs. magnitude) and the stimulus attribute (number vs. digit) switched, and trials on which only one criterion switched (e.g., from digit parity to magnitude discrimination). Although partial switches in principle allowed for old control representations to be reused, no difference was observed between full and partial switches—indicating that, following a task switch, *all* task-set components are reset. Finally, in a study very similar to Kleinsorge (2004); Vandierendonck et al. (2008) (see also Kleinsorge and Heuer, 1999) observed partial repetition *costs*, that is, partial switches took actually more time to be implemented than full switches. Kleinsorge explained these findings by assuming a hierarchical organization of task sets, according to which having to change task set components situated earlier in the stimulus-response chain (e.g., identification criteria) would trigger a switch in all subsequent criteria (e.g., S-R mapping rules). In summary, the available literature suggests that following a task-switch, task-sets are sometime reset, sometimes switched, and sometimes reused. It should be borne in mind, however, that the various studies used (i) different paradigms and (ii) different types of switches. These differences will be discussed in more detail in the General Discussion.

### Resetting, switching or reusing task-set components

Depending on what tasks precisely vary across trials, task switching could necessitate changes of either all components (full switches) or only those components in which the two tasks differ (partial switches). To illustrate, consider a paradigm in which stimulus displays consist of many identical items with one of them (the singleton target) being different in either color or orientation from the rest. Participants are to, on different trials, either simply detect the presence vs. absence of the singleton target or discriminate its exact features, with a cue, presented prior to the display onset, announcing what task (detection or discrimination) is to be performed. Independently of the task sequence, the task-relevant dimension can also repeat or change. The dimension in which the singleton target is defined, although not informative about the response to be performed (i.e., knowing the dimension would not specify the exact response!), would be informative for both spatial-attentional selection and post-selective identification processes.

Concerning target selection, the available evidence suggests that when the dimension repeats across trials, spatial selection is speeded relative to dimension changes, as elaborated in the Dimension-Weighting Account of Müller and colleagues (e.g., Found and Müller, 1996; Müller and Krummenacher, 2006; Müller, 2010). Since both singleton detection and singleton discrimination tasks would require spatial selection, spatial selection processes for *both* tasks would be sensitive to the singleton dimension. Accordingly, dimension repetition effects (DREs) would be expected across (task repetition and switch) trials of both detection and discrimination tasks.

In contrast to the shared spatial selection, post-selective identification processes should differ between the two tasks. On the one hand, fast and accurate singleton *detection* can be achieved by simply determining the presence/absence of a singleton, while information about what the singleton features precisely are is not strictly necessary for performing the task. Consistent with this, there is evidence that the response-irrelevant singleton features are not processed up to the level at which they become available for explicit report (Müller et al., 2004). On the other hand, encoding singleton features from a particular dimension is critical for the singleton *discrimination* task. With these differential task requirements in mind, it is likely that post-selective identification processes differ between tasks: the singleton dimension is important for identification in the discrimination, but not in the detection task (see Töllner et al., 2012b, for supporting EEG data).

Differences between detection and discrimination tasks would determine what *can be* and *is* reused across trials. Following performance of a target-present detection task, the dimension should have been encoded and thus be available for reuse only for spatial selection. Following a discrimination task, the dimension should have been encoded in and thus be available for reuse for both the spatial selection and post-selective identification processes. Thus, the task on trial *n* − 1, or *prime* trial, determines what is *available* for reusing. By contrast, the task on trial *n*, or *probe* trial, determines what *is* reused: in the detection task, reusing dimension information would facilitate just spatial selection, while in discrimination task reusing dimension information would facilitate both selection and identification processes. Consequently, on the hypothesis of agglomerated task sets, comparable DREs would be expected for detection → detection and detection → discrimination sequences, because across these sequences, only spatial selection criteria are available and reused. By contrast, following performance of the discrimination task, both spatial selection and identification criteria would be available for reuse, but they would be reused only on the current discrimination trial, predicting stronger DREs for discrimination → discrimination relative to discrimination → detection sequences.

In contrast to the notion of agglomerated task sets, that of integrated task sets would predict that following *any* switch, the task set would be reset; accordingly, there should not be a difference between full and partial switches. Finally, the notion of hierarchical task sets would predict a reversal of DREs, that is: switching from, e.g., detection to discrimination, would also switch the expected dimension, resulting in worse performance following dimension repetitions relative to dimension switches.

### ERP components sensitive to the spatial selection and post-selective identification components

In the paradigm described above, preparatory adjustments with regard to the task-relevant (singleton) dimension are not possible since the task cue is not dimension-specific^1^. Consequently, the analyses of partial-switch effects for spatial selection and post-selective identification in the EEG domain have focused on areas and ERP components sensitive to the implementation of these processes.

As an index of spatial-attentional selection, parameters of the Posterior-Contralateral-Negativity (PCN, or N2-posterior-contralateral^2^) have been analyzed. This component manifests as an increased negativity at posterior scalp electrode sites contralateral to the singleton position, relative to ipsilateral electrode sites, in the 175–300-ms time range post-stimulus. PCN parameters are considered to reflect the dynamics of spatial selection processes (Luck and Hillyard, 1994; Eimer, 1996; Töllner et al., 2011). Importantly, PCN latencies are shorter for dimension repetitions relative to changes (Töllner et al., 2008). On the assumption of agglomerated task sets, substantial DREs are expected for both task repetitions and switches. By contrast, the assumption of integrated tasks sets would predict no DRE across task switches.

The second component of interest was the Sustained Posterior Contralateral Negativity (SPCN), manifesting as an increased negativity over posterior electrodes contralateral to the target, relative to ipsilateral electrodes, starting from 350–400-ms post-stimulus. The SPCN component is considered to be sensitive to the processing demands following stimulus selection (Jolicoeur et al., 2008). Importantly, the SPCN is weaker (Mazza et al., 2007; Töllner et al., 2013) in tasks that do not necessarily require perceptual analysis following stimulus selection (e.g., singleton detection task), and more prominent (ibid.) in tasks that require stimulus analysis up to the feature levels (e.g., in singleton discrimination task). Thus, the available evidence predicts a stronger SPCN for the discrimination task, which requires explicit feature discrimination, relative to the detection task, in which post-selective processing is not necessary for an accurate response. Furthermore, the hypothesis of agglomerated task sets predicts stronger DREs in the SPCN time range on trials preceded by (a trial with) the discrimination task, in which post-selective identification should be sensitive to the dimensional identity of the singleton, relative to trials preceded by the detection task, for which it is sufficient to determine that the selected item is a singleton, without necessarily identifying its precise dimension or feature properties.

## Methods

### Participants

Sixteen males (mean age 29 years, 2 left-handed), all with normal color vision and normal or corrected-to-normal visual acuity, participated in the experiment for monetary compensation. All participants had extensive experience with psychophysical tasks and all were naïve as to the purpose of the experiment. Due to excessive eye blinking, two participants were excluded from the analyses.

### Apparatus

Stimuli were presented on a 17″ CRT monitor with a 1024 × 768 pixels resolution and an 85-Hz refresh rate. Custom written C^++^ code controlled stimulus presentation and recorded responses. The experiment was conducted in a dimly lit, acoustically and electromagnetically shielded room. Head-to-monitor distance was 60 cm.

### Stimuli and procedure

**Figure 1:**
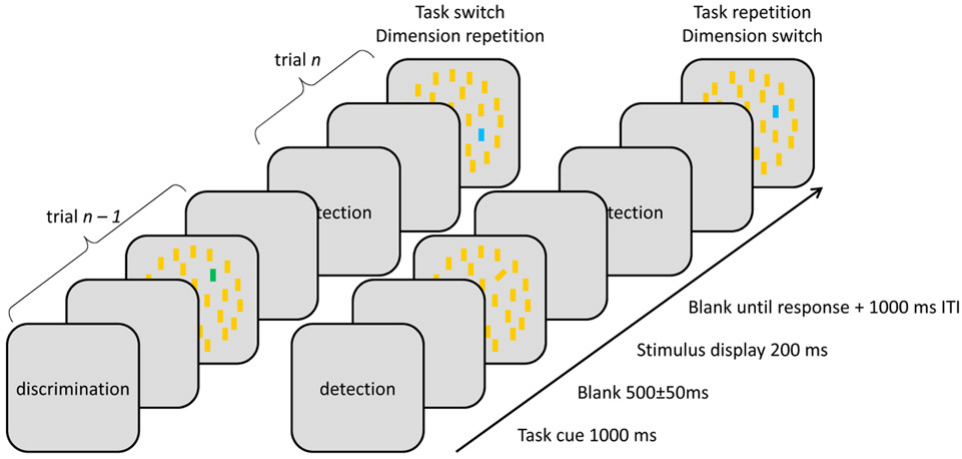
**Illustration of stimulus displays across different inter-trial sequences**.

Stimulus displays (Figure 1) were presented on a gray background (19 cd/m^2^, CIE *x* = 0.292, *y* = 0.307) and consisted of 38 vertical yellow (0.388, 0.520) bars arranged around three concentric—inner, middle, and outer—circles with 8, 12, and 18 items, respectively. Single bars subtended 0.4 × 1.9° of visual angle, and the diameters of the three circles were 5, 10, and 15°, respectively. On target-present trials, one of the bars on the middle circle (excluding the two positions along the vertical meridian) was replaced by either a blue (0.275, 0.541) or a green (0.211, 0.263) *color* singleton target or a right-titled (12° clockwise from vertical) or left-tilted (12° counter-clockwise) *orientation* singleton target, matched in luminance to the distractor bars (68 cd/m^2^).

Every trial started with a task cue (i.e., the word “detection” or “discrimination”) shown for 1000 ms, followed by a 500 ± 50-ms blank screen. Next, the stimulus display appeared for 200 ms, followed by a blank screen until response. In case of response errors, the standard intertrial interval (1000 ms) was doubled. Responses were given via pressing the left and right mouse keys using the left- and right-hand thumbs, respectively. Stimulus displays were identical for both tasks, the difference being that, in the detection task, participants were required to discern the presence (on 60% of detection task trials) vs. the absence of a singleton target by pressing the corresponding response key with two possible S-R mappings: (i) target-present → R_1_, -absent → R_2_ and, respectively, (ii) target-absent → R_1_, -present → R_2_. In the discrimination task, a singleton was always present, with participants having to report the feature that distinguished it from distractors (blue, green, left-, and right tilted). Same-dimension singletons (e.g., blue and green) required different responses (e.g., R_1_ and R_2_), while singletons from different dimensions (e.g., blue and left-tilted) were mapped to a same response, resulting in four possible S-R mappings: (i) blue or left-tilted → R_1_, green or right-tilted → R_2_, (ii) green or left-tilted → R_1_, blue or right-tilted → R_2_, (iii) blue or right-tilted → R_1_, green or left-tilted → R_2_, and (iv) green or right-tilted → R_1_, blue or left-tilted → R_2_. The two possible S-R mappings in the detection task and the four S-R mappings in the discrimination task yielded eight different S-R mapping combinations, which were counterbalanced across participants.

The task (detection vs. discrimination) and the target's dimension (color vs. orientation) were randomly selected on every trial, resulting in four task sequences (detection on both prime and probe trials; discrimination on both trials; detection on prime, discrimination on probe trial; and discrimination on prime, detection on probe trial) and two dimension sequences (repetition/change) across consecutive trials. Relevant dimensions were sampled with equal probability, however, in order to ensure comparable numbers of target-present trials across the two tasks, the detection task was made more frequent (3:2 ratio).

Prior to experiment proper, participants received 1–4 practice blocks (80 trials per block); practice was terminated once a participant achieved ≤ 10% errors per block. All participants met the criterion after 2–4 blocks. Following practice, participants completed 1920 trials (in ca. 3 h), split in two equal-length sessions with a 15–30 min break in between.

### EEG recording and analyses

The EEG was sampled at 1 KHz using Ag/AgCl active electrodes (actiCAP system; Brain Products, Munich) from 64 scalp sites, arranged according to the 10–10 System (American Electroencephalographic Society, 1994), and amplified using BrainAmp amplifiers (BrainProducts, Munich) with a 0.1–250-Hz bandpass filter. Impedances were kept below 5 kΩ. Electrodes were online referenced to FCz and re-referenced offline to averaged mastoids. Electrodes placed at the outer canthi of the eyes and the superior and inferior orbits monitored blinks and eye movements. Non-stereotyped noise was removed by visual inspection, followed by high-pass filtering using a Butterworth infinite impulse response filter at 0.5 Hz (24 dB/Oct). An infomax independent-component analysis was run to identify and remove effects of eye movements and blinks. Next, continuous EEG was epoched into −200–600 ms segments time-locked to stimulus display onset. Baseline correction was performed based on the −200–0 ms pre-stimulus interval. Target-absent trials, trials preceded by a target-absent trial, error response trials, as well as trials preceded by an error were excluded from the analyses. Finally, trials with (i) signals exceeding ±60 μV, (ii) bursts of electromyographic activity (permitted maximal voltage steps/sampling point of 50 μV), or (iii) activity lower than 0.5 μV within intervals of 500 ms (indicating dead channels) were removed from further analyses on an individual channel basis. The remaining trials (mean = 111 trials per participant per condition, *SD* = 5 trials) were sorted according to experimental conditions, and averaged on an individual-channel basis.

### Data analyses

Analysis of EEG signals focused on two event-related potentials (ERPs). The ERP components were quantified by subtracting ERPs measured at lateral parieto-occipital electrodes (PO7/PO8) ipsilateral to the target's location from contralateral ERPs. The PCN peak latencies and amplitudes were defined per participant as the maximum negative-going deflection in the time period 170–270 ms post-stimulus. SPCN amplitudes were defined as the average of the time window the 430–510-ms post-stimulus.

Mean RTs, error rates, PCN peak latencies and amplitudes, and SPCN mean amplitudes were computed for correct target-present probes for which the primes were also correct target-present trials. Dependent measures were submitted to repeated-measures ANOVAs (RANOVAs) in three different analyses. First, overall task differences and TSCs were assessed in a RANOVA with main terms for (i) task on *probe* trial (detection vs. discrimination) and (ii) task sequence (repetition vs. change) across prime and probe trials. The second analysis focused on indices of re-using processes across trials, that is, on dimension-repetition effects. Because re-use is expected to co-vary with what is *available* for re-using, which is determined on prime trials, the second set of analyses used a RANOVA with main terms for (i) task on *prime* trial, (ii) task on *probe* trial, and (iii) dimension sequence (repetition vs. change) across trials. Third, effects of response sequence on mean RTs and error rates were analyzed. Because only a small number of trials were available per cell for this analysis, the corresponding ERPs could not be examined.

## Results

### Analyses of overall task differences

#### Behavioral results

Inspection of the overall mean RTs and error rates (shown in Table 1) revealed performance to be faster and more accurate for the detection task (mean RTs = 594 ms, mean error rate = 5.2%) than for the discrimination task (670 ms, 6.5%). Furthermore, task repetitions (598 ms, 4.5%) yielded faster and more accurate performance relative to task changes (666 ms, 7.1%), indicating substantial TSCs for both RTs and error rates (TSC_RT_ = 68 ms, TSC_errors_ = 2.6%). Finally, switching from discrimination to detection incurred greater TSCs (81 ms, 3%) than switching from detection to discrimination (57 ms, 2.3%).

**Table 1:** **Mean RTs (SE_M_) and percentage of errors (SE_M_) on probe trials for the two different tasks, dependent on the task sequence**.

**Task on probe trial**	**Task sequence**	**RTs**	**Errors**
Detection	Repetition	553 (23)	3.7% (0.4)
	Change	634 (30)	6.7% (0.8)
Discrimination	Repetition	642 (27)	5.3% (1)
	Change	698 (32)	7.6% (1)

These observations were confirmed by RANOVAs of the mean RTs and error rates with main terms for task on probe trial (detection vs. discrimination) and task sequence (task- repetition vs. -change). The analysis of the mean RTs proved main effects of task, *F*_(1,13)_ = 36.44, *p* < 0.01, η^2^_*p*_ = 0.74, task sequence, *F*_(1,13)_ = 18.65, *p* < 0.01, η^2^_*p*_ = 0.60, as well as their interaction, *F*_(1,13)_ = 6.18, *p* < 0.05, η^2^_*p*_ = 0.32, to be significant. The RANOVA of the mean error rates yielded likewise a significant main effect of task sequence, *F*_(1,13)_ = 20.94, *p* < 0.01, η^2^_*p*_ = 0.62, without, however, any of the other effects reaching significance (all *F* < 2.17, all *p* > 0.16).

#### ERP results

**Figure 2:**
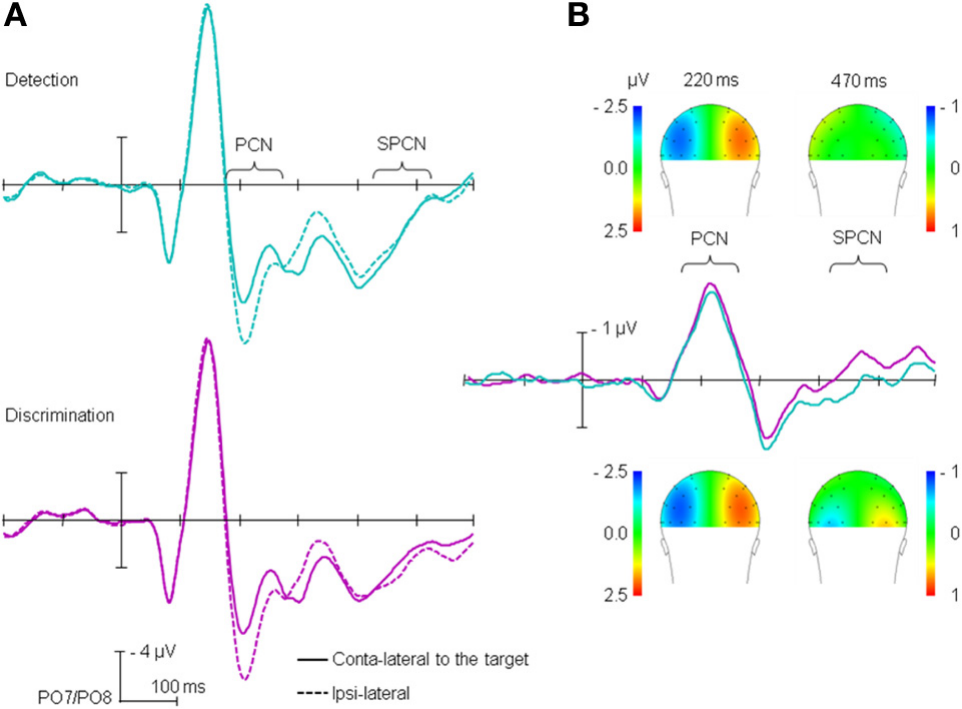
**Stimulus-locked ERPs: (A) for the ipsi-and contralateral electrodes relative to the target position, separately for the detection and discrimination tasks; (B) difference between contra- and ipsilateral electrodes.** For the purpose of the presentation, data are presented filtered with 30-Hz low-pass filter.

The stimulus-locked ERP waves obtained for the detection and discrimination tasks are shown on Figure 2. Figure 2A depicts the time course separately for electrodes contra- and ipsilateral to the target position, averaged across target positions^3^ (left vs. right), while Figure 2B depicts the difference between contra- and ipsilateral electrodes. As can be seen from Figure 2B, substantial lateralization effects, confirmed by *t*-tests against zero, were observed in the PCN time range (170–270 ms) for both the detection (−2.17 μV, *p* < 0.01) and the discrimination task (−2.41, *p* < 0.01). Similar to what we already reported in Töllner et al. (2012a), PCN amplitude was higher for the discrimination than for the detection task, with comparable PCN latencies across the two tasks (221 and 222 ms for detection and discrimination, respectively). The RANOVA of the PCN latencies with main terms for (i) task on probe trial and (ii) task sequence yielded no significant effects (all *F* < 1). The RANOVA of the PCN amplitudes yielded a significant main effect of task, *F*_(1,13)_ = 6.61, *p* < 0.05, η^2^_*p*_ = 0.43; the main effect of task sequence non-significant (*F* < 1); the task × task sequence interaction approached significance, *F*_(1,13)_ = 3.70, *p* = 0.08, η^2^_*p*_ = 0.22), owing to the fact that switching task tended to increase the PCN amplitude for the detection task (−2.12 μV and −2.22 μV for detection → detection and discrimination → detection sequences, respectively), while tending to decrease the amplitude for the discrimination task (−2.52 and −2.29 μV for discrimination → discrimination and detection → discrimination sequences, respectively).

Furthermore, as Figure 2B shows, and as confirmed by *t*-tests against zero, lateralization effects in SPCN time range (430–510 ms) were observed for the discrimination task (mean amplitude = −0.36 μV, *p* < 0.01), but not for the detection task (0.14 μV, *p* = 0.18). A RANOVA of the SPCN mean amplitude with main terms for task and task sequence revealed the main effect of task to be significant, *F*_(1,13)_ = 72.74, *p* < 0.01, η^2^_*p*_ = 0.85, with no other effects reaching significance (all *F* < 1).

### Analyses of re-using control settings across tasks

#### Behavioral results

**Figure 3:**
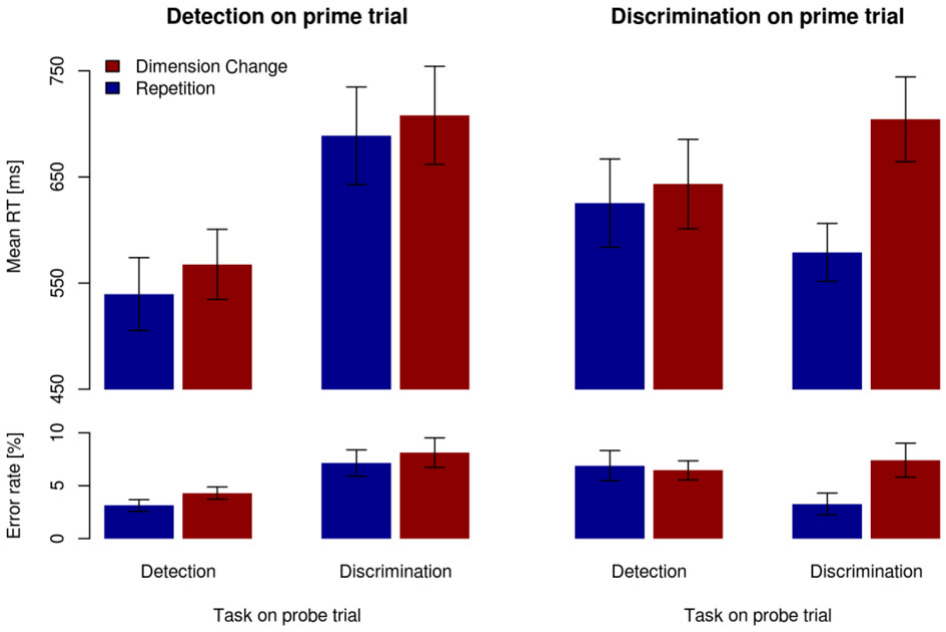
**Mean RTs (upper panels) and error rates (lower panels) for the different task sequences, separately for dimension repetitions (blue bars) and changes (red bars).** Vertical lines denote ±1SEM.

Figure 3 depicts the mean RT and error rate for a given task on the probe trial (detection, discrimination) dependent on the task on the prime trial (detection, discrimination), separately for dimension repetitions and changes (dimension sequence). As can be seen, RTs were faster for dimension repetitions (blue bars) than for dimension changes (red bars), in all conditions; error rates followed a similar pattern. These observations were confirmed by a RANOVA of the mean RTs, which revealed all three main effects (all *F* > 6.18, all *p* < 0.05), all two-way interactions (all *F* > 18.17, all *p* < 0.01), and the three-way interaction between task on prime trial, task on probe trial, and dimension sequence, *F*_(1,13)_ = 21.27, *p* < 0.01, η^2^_*p*_ = 0.62, to be significant. An analogous RANOVA of the error rates yielded the following significant effects: main effect of dimension sequence, *F*_(1,13)_ = 11.39, *p* < 0.01, η^2^_*p*_ = 0.47; task on probe trial × dimension sequence interaction, *F*_(1,13)_ = 33.88, *p* < 0.05, η^2^_*p*_ = 0.28; task on prime trial × task on probe trial interaction, *F*_(1,13)_ = 20.94, *p* < 0.01, η^2^_*p*_ = 0.62; and task on prime trial × task on probe trial × dimension sequence interaction, *F*_(1,13)_ = 8.16, *p* < 0.5, η^2^_*p*_ = 0.39.

To further investigate the significant three-way interactions, separate RANOVAs were run dependent on the specific task on prime trials (detection and, respectively, discrimination), with main terms for task on probe trial and dimension sequence. With the detection task on prime trials (left-hand panels in Figure 3), the analysis of the (probe-trial) RTs revealed both main effects to be significant: task on probe trial [145-ms difference between discrimination and detection tasks, *F*_(1,13)_ = 34.04, *p* < 0.01, η^2^_*p*_ = 0.72] and dimension sequence [23-ms difference between dimension changes vs. -repetitions, *F*_(1,13)_ = 22.00, *p* < 0.01, η^2^_*p*_ = 0.63]; the interaction between the two was far from significance (*F* < 1). The RANOVA of the error rates revealed a significant main effect of task on probe trials [3% difference between discrimination and detection, *F*_(1,13)_ = 16.68, *p* < 0.01, η^2^_*p*_ = 0.56], and a marginally significant main effect of dimension sequence, with dimension repetitions yielding 1.1% more accurate performance than dimension changes, *F*_(1,13)_ = 4.35, *p* = 0.06; the interaction between the two was far from significance (*F* < 1).

With the discrimination task on prime trials (right-hand panels in Figure 3), a RANOVA of the (probe-trial) RTs revealed the main effect of dimension sequence, *F*_(1,13)_ = 36.32, *p* < 0.01, η^2^_*p*_ = 0.81, and the task on probe trial × dimension sequence interaction, *F*_(1,13)_ = 32.01, *p* < 0.01, η^2^_*p*_ = 0.71, to be significant. The interaction was caused by the dimension repetition (vs. change) effect being much larger for discrimination → discrimination sequences (125 ms) than for discrimination → detection sequences (18 ms). For the errors (on probe trials), the main effect of dimension sequence proved significant, *F*_(1,13)_ = 7.67, *p* < 0.05, η^2^_*p*_ = 0.37, with accuracy being 1.4% higher for dimension repetitions relative to changes. The task on probe trial × dimension sequence interaction was also significant, *F*_(1,13)_ = 8.31, *p* < 0.5, η^2^_*p*_ = 0.40, with a stronger DRE for the discrimination relative to the detection task on the probe trial (3.2 vs. −0.3%, respectively).

In summary, significant DREs were observed in all experimental conditions. When the task on the prime trial required simple target detection, DREs were comparable for detection and discrimination tasks on the probe trial. By contrast, with the task on prime trial required target discrimination, stronger DREs were observed for the discrimination task, relative to the detection, to be performed on the probe trial.

#### ERP results

**Figure 4:**
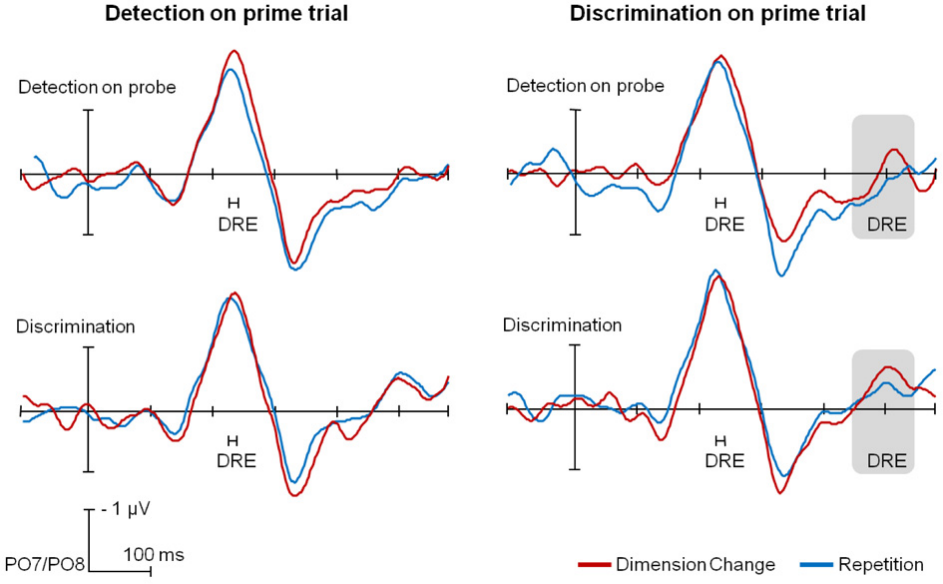
**Group-averaged time course of PCN and SPCN components for the different task sequences, separately for dimension repetitions (blue) and changes (red).** Significant dimension repetition effects for the peak PCN latency and the mean SPCN amplitude are indicated. For the purpose of presentation, a 30-Hz low-pass filter was applied; data analyses were performed over individual, unfiltered data.

Lateralized ERPs are depicted in Figure 4 for the probe trials, separately for the different tasks on prime and on probe trials, as well as across different dimension sequences. As can be seen from Figure 4, the PCN latency was delayed for dimension changes (red lines) relative to dimension repetitions (blue lines), in all conditions. In the SPCN time range DREs were evident only for (probe) trials following the discrimination task.

#### PCN analyses

For (probe) trials preceded by the detection task (left-hand side of Figure 4), a RANOVA of the PCN peak latencies revealed only a significant main effect of dimension sequence, with dimension repetitions being 17 ms faster than dimension changes, *F*_(1,13)_ = 15.41, *p* < 0.01, η^2^_*p*_ = 0.54; no other effects reached significance (all *F* < 1). An analogous RANOVA for trials preceded by the discrimination task (right-hand side) also yielded only a significant main effect of dimension sequence [14-ms DRE, *F*_(1,13)_ = 5.09, *p* < 0.05, η^2^_*p*_ = 0.23], with no other effects reaching significance (all *F* < 1).

#### SPCN analyses

With the task on the prime trial requiring target detection, analysis of the (probe-trial) mean SPCN amplitudes revealed only a significant main effect of task on probe trial, with an overall stronger SPCN component for the discrimination relative to the detection task (−0.37 vs. 0.18 μV), *F*_(1,13)_ = 25.70, *p* < 0.01, η^2^_*p*_ = 0.66; no other effects reached significance (all *F* < 1.94, all *p* > 0.18). With the discrimination task on the prime trial, an analogous analysis also yielded a significant main effect of task on probe trial, *F*_(1,13)_ = 30.16, *p* < 0.01, η^2^_*p*_ = 0.70, with the significant SPCN for the discrimination relative to the insignificant SPCN for the detection task (−0.36 vs. 0.10 μ V). Importantly, the main effect of dimension sequence was also significant, *F*_(1,13)_ = 5.33, *p* < 0.05, η^2^_*p*_ = 0.29, with the SPCN amplitude being stronger for dimension changes relative to dimension repetitions (−0.23 vs. −0.02 μ V); the interaction task on probe trial x dimension sequence was far from significance (*F* < 1).

In summary, analyses of the ERPs revealed longer PCN peak latencies for dimension changes vs. repetitions independently of the task on prime or probe trial; SPCN mean amplitudes were overall larger for the discrimination than for the detection task on probe trial, while significant DREs in the SPCN time interval were observed only for trials preceded by the discrimination task.

### Analyses of response-sequence effects

As can be seen from Table 2, mean RTs were overall fastest for full repetitions (same task, dimension, and response), intermediate for partial repetitions, and slowest for full changes. By contrast, the errors varied as a function of task sequence. Importantly, though, there was no evidence that partial repetitions resulted in less accurate performance relative to full changes.

**Table 2:** **Mean RTs (SE_M_) and percentage of errors (SE_M_) across different task-, dimension- and response^a^ sequences**.

**Intertrial sequence of**		**Detection → Discrimination**		**Discrimination → Detection**		**Discrimination → Discrimination**	
**Dimensions**	**Responses**	**RTs**	**Errors**	**RTs**	**Errors**	**RTs**	**Errors**
Change	Change	715 (46)	9.6 (1.5)	641 (40)	8.1 (1.0)	708 (40)	5.9 (1.3)
	Repetition	703 (47)	6.5 (1.3)	646 (45)	4.7 (0.8)	701 (40)	8.7 (2.1)
Repetition	Change	713 (48)	4.2 (1.0)	627 (40)	8.7 (2.0)	587 (28)	1.6 (0.5)
	Repetition	667 (44)	9.5 (1.7)	624 (43)	5.2 (1.0)	558 (27)	4.9 (1.5)

a Response sequence analysis for detection → detection task sequence was omitted because it always involved a response repetition.

These observations were confirmed by three-ways RANOVAs (task- × dimension- × response sequence) of the mean RTs and error rates with a focus on the main effect of and interactions involving response sequence. The RT analysis showed the main effect of response sequence to be significant, *F*_(1,13)_ = 6.41, *p* < 0.05, η^2^_*p*_ = 0.33, as well as the interaction of this factor with dimension sequence, *F*_(1,13)_ = 9.04, *p* < 0.01, η^2^_*p*_ = 0.41, owing to a larger response repetition benefit when the dimension repeated (30 ms, *p* < 0.01) rather than changed (5 ms, *p* = 0.49). The task sequence × response sequence interaction was marginally significant, *F*_(2,26)_ = 1.67, *p* = 0.07, η^2^_*p*_ = 0.18, suggestive of a larger response repetition benefit for the discrimination task (29 ms, *p* < 0.01, and 23 ms, *p* < 0.01, for detection → discrimination and discrimination → discrimination sequences, respectively) relative to the detection task (−1 ms, *p* = 0.93). The three-way interaction did not reach significance, *F* = 1.67, *p* = 0.27.

Concerning the error analysis, the main effect of response sequence was non-significant (*F* < 1). However, response sequence interacted with dimension sequence, *F*_(1,13)_ = 18.43, *p* < 0.01, η^2^_*p*_ = 0.59, and task sequence, *F*_(2,26)_ = 18.52, *p* < 0.01, η^2^_*p*_ = 0.59; and the response- × dimension- × task sequence interaction was significant, *F*_(2,26)_ = 8.55, *p* < 0.01, η^2^_*p*_ = 0.40. *Post-hoc* analyses revealed the pattern of response sequence effects (repetition benefit vs. cost) to vary across task and dimension sequences. For discrimination → detection task sequences, dimension repetitions were associated with response repetition *costs* (−5.4%, *p* < 0.01), whereas dimension changes yielded response repetition *benefits* (4.1%, *p* < 0.01). By contrast, discrimination → detection task sequences resulted in response repetition benefits (3.5%, *p* < 0.01) independently of dimension sequence. Finally, discrimination → discrimination task sequences were associated with response repetition costs (−2.9%, *p* < 0.05) independently of dimension sequence.

In summary, analyses of response sequence effects on mean RTs showed either response repetition benefits or no effects of response sequence. This finding is at variance with hierarchical task sets, which predict response repetition costs following a task or dimension change. Integrated task sets, which predict no response sequence effects following a task or dimension switch, also account poorly for the present findings. The results for error rates were less consistent across task and dimension sequences, with different patterns of response sequence effects across different experimental conditions.

## Discussion

The present findings demonstrate that, consistent with “agglomerated task sets,” it is possible to reuse control settings across different tasks, and that the reusing is associated with distinct ERP components, depending on precisely which task set component (selection vs. identification criteria) is reused across tasks. In particular, switching tasks was revealed to be easier when the task-relevant dimension repeated relative to when it changed, as indexed by DREs on task switch trials. The notion of agglomerated task sets is fully compatible with existing computational models of cognitive control (Logan and Gordon, 2001; Meiran et al., 2008). Both these accounts postulate several independent parameters influencing processes of selection, identification, and, respectively, responding. Importantly, since these parameters are independent, it is plausible that they can also be adjusted independently—which is the core assumption of the agglomerated-task-sets hypothesis. The compatibility with the computational models of cognitive control emphasizes another property of agglomerated task sets: their computational efficiency.

Note though that the DREs reported presently are consistent not only with agglomerated task sets, but also with two, relatively strong alternatives, namely: (i) switching between the detection and discrimination conditions did not involve a task switch, and (ii) the DREs are not dimension-specific, but rather feature- or response-specific. The former alternative would postulate that the two tasks used in the present study were effectively one task, in which participants always performed feature discrimination, but, depending on the cue, selected different responses. The latter alternative would imply that the substantial reusing of spatial selection and identification criteria (as revealed in the dimension-repetition effects) was not indicative of the reuse of task set components in general, but rather of the reuse of stimulus-response associations encountered on the previous trial (Hommel et al., 2001; Dreisbach et al., 2006, 2007).

The present findings argue against the hypothesis that no task switching took place in our paradigm. First, in the present study, substantial differences in mean RTs and errors rates were observed between the detection and discrimination tasks, suggesting that the two conditions were performed differently. Behavioral differences were accompanied by differences in the ERPs: a reliable SPCN component was observed for the discrimination, but not for the detection task. Taken together, these findings suggest that solving the detection and, respectively, discrimination tasks involved the use of different task sets. Second, switching between the two conditions incurred substantial switch costs. Importantly, the switch cost magnitude differed, with stronger costs for discrimination → detection relative to detection → discrimination sequences. The asymmetry in the switch costs—with switching to an easier, or dominant, task (presently, detection) being more effortful than switching to a more difficult task (presently, discrimination)—has frequently been reported in task-switching literature (Allport et al., 1994; Wylie and Allport, 2000) and interpreted as an index of the interference between two concurrent task sets. On this background, it is likely that switching between the two tasks in the present study indeed involved task switch processes.

The second alternative explanation posits that DREs critically rely on repetitions of full stimulus-response episodes across trials, rather than on more abstract criterion repetitions. Studies investigating the role of S-R repetitions have typically found performance to be very good on full-repetition trials and worse on partial-repetition trials, that is, when either the stimulus or the response changed, while the other property repeated. Somewhat counter-intuitively, performance is typically *better* on full switches, that is, when both stimulus and response change, compared to partial repetitions (Hommel, 1998, 2005; Töllner et al., 2008; Zehetleitner et al., 2012). Applied to the present study, if DREs were actually S-R repetition effects, then changing the dimension and repeating the response (i.e., partial repetitions) should have resulted in worse performance relative to changing both the dimension and the response (full repetitions). However, analyses of response sequence effects showed this not to be the case, arguing that the DREs reported presently are not reducible to S-R repetition effects.

That partial-repetition costs were not prominent in the present study does not necessarily imply that the processes generating these effects were inactive; rather, the paradigm and dependent measures may not have been sensitive to these processes. Previous work investigating electrophysiological correlates of DREs in a single task paradigm (Töllner et al., 2008) revealed partial repetition costs to correlate with ERP markers that were independent of the ERP markers for dimension and, respectively, response sequence effects. Importantly, the markers of partial-repetition costs were not investigated in the present study. Additionally, in a recent study of partial-repetition costs (Zehetleitner et al., 2012), numerical simulations showed that three different sequence-sensitive mechanisms (dimension-, S-R mapping-, and motor-response-specific) combine and can produce any possible RT pattern, that is, with or without manifest partial repetition costs. Given this, until the boundary conditions for partial-repetition costs to arise are fully understood, it remains possible that the present paradigm simply did not meet these conditions.

### Direction of partial-switch effects: reusing, resetting, or switching

While the present study, together with several previous investigations (Arrington et al., 2003; Rangelov et al., 2011, 2012), showed that reusing shared task set components across different tasks is possible, findings to the contrary have also been reported. In particular, Vandierendonck et al. (2008) failed to find any partial-switch effects, while Kleinsorge and colleagues (1999, 2004) found partial-switch *costs*, relative to full switches, rather than benefits as reported presently. How can these disparate findings be reconciled?

A notable difference between the paradigms used in these investigations and the present study is in the stimulus material and the cueing procedure employed. More precisely, the previous studies used stimuli that were ambiguous with regard to both task-relevant stimulus attributes and identification criteria. Thus, cueing of both the relevant stimulus property (e.g., number or digit value in Vandierendonck et al., 2008) and identification criteria (parity vs. magnitude) was necessary on every trial (e.g., the string “number odd/even” served as a cue). This procedure produced partial overlap between cue strings on partial switches (e.g., “number odd/even” → “digit odd/even”) and no overlap on full switches (e.g., “number smaller/greater” → “digit odd/even”). As has been shown by several studies (Logan and Schneider, 2006; Schneider and Logan, 2009), overlapping cues activate competing task sets, resulting in negative interference on partial-switch trials. As a consequence, any benefits from reusing shared control representations might have been masked by interference effects from overlapping cues, resulting in either insignificant partial-repetition effects or even partial-repetition costs. By contrast, the present study used cues that did not overlap between the different tasks. Furthermore, the task-relevant dimension was unambiguously specified by the stimulus displays themselves, so no dimension cues were necessary. Consequently, in the present experiment, the negative interference effects would have been minimal—and, correspondingly, partial-repetition effects turned out significant.

### Electrophysiological evidence for separable control mechanisms

In contrast to the mixed behavioral findings regarding the direction of partial-switch effects, investigation of the ERP components related to the spatial selection and post-selective processing task components offers more conclusive findings. By assuming relative autonomy of the control systems for the different cognitive processes, “agglomerated task sets” predict the existence of *multiple*, relatively independent sources of DREs. Consistent with the prediction, EEG analyses revealed *multiple* ERP components to be sensitive to dimension sequences: dimension changes (relative to repetitions) resulted in longer PCN latencies as well as larger SPCN amplitudes.

Analyses of the PCN latencies showed dimension changes (relative to repetitions) to be associated with longer latencies independently of the task sequence (see also Töllner et al., 2008, reporting similar findings in a single-task paradigm). This finding can be explained by assuming that both the detection and discrimination tasks required spatial selection processes, governed by a task set component sensitive to dimension sequences. Dimension repetitions would permit reusing control settings from the previous trial, generating DREs for the PCN latency. Importantly, on the agglomerated-task-set hypothesis, reusing settings is possible even across tasks that differ in other task set components—as evidenced by the present finding of DREs across task switches for the PCN timing.

Analyses of the SPCN amplitudes showed, consistent with our predictions, a more pronounced SPCN for discrimination relative to detection task trials. Apparently, the SPCN amplitude increases continuously with increases in demands for post-selective perceptual processing, from no SPCNs in singleton detection (present study) and singleton localization tasks (Mazza et al., 2007) through singleton discrimination tasks (present study) to strong SPCNs in compound-search tasks (Mazza et al., 2007; Töllner et al., 2013), in which one target dimension (e.g., color) is selection-relevant, whereas another dimension (e.g., orientation) is response-relevant.

Furthermore, the SPCN amplitude was sensitive to dimension sequence, but only on trials following the discrimination task. This finding is consistent with the conceptual analysis of the task sets for the detection and, respectively, the discrimination task and the functional interpretation of the SPCN component. Conceptual analysis of the detection task suggested that the singleton dimension is not mandatorily encoded in the post-selective identification settings (for the detection task) and it would not be available for reuse on the following trial, predicting no DRE for the SPCN component following detection task trials. By contrast, post-selective identification in the discrimination task necessarily involved determining the singleton dimension, predicting DREs for the SPCN component following trials of the discrimination task, consistent with the results of the present study.

The electrophysiological data are especially informative about the notion of a generalized switching mechanisms [as proposed by Kleinsorge and Heuer (1999)], according to which one criterion switch enforces a switch in all criteria, rather than simple re-setting. If generalized switching were operating as a default task switch mechanism, then reconfiguring the spatial selection component should have triggered a switch in the post-selective identification component as well. This would predict an obligatory coupling between DREs for the PCN and SPCN parameters—a prediction that is not supported by the present findings. Thus, in summary, the present ERP findings are fully consistent with, and yet independent of the behavioral findings of DREs across task switches, in their support for the notion of agglomerated task sets.

## Conclusions

The present study investigated the nature of task-set representations, defined as a set of criteria for spatial selection, post-selective identification, and S-R mapping rules. Two alternatives were considered: task sets as integral representations and task sets as agglomerations of relatively autonomous control settings guiding different processing stages. The key property of the “agglomerated-task-set” hypothesis is that following a task switch, different control instances can be reconfigured independently of each other, permitting reusing some of the control settings across different tasks. By contrast, the hypothesis of “integrated task sets” predicted no partial-switch effects, as changing any task set component would either reset all settings, or trigger a full switch. Consistent with agglomerated task sets, our findings demonstrate substantial partial-switch effects, operationalized as DREs. Most importantly, evidence of DREs over PCN latencies and SPCN amplitudes, two functionally distinct ERP components, further supports the idea of task sets as a collection of autonomous control instances governing processes of spatial selection and perceptual/symbolic analysis, respectively.

### Conflict of interest statement

The authors declare that the research was conducted in the absence of any commercial or financial relationships that could be construed as a potential conflict of interest.

## Footnotes

^1^Note that most of the previous EEG studies on task switching used paradigms in which preparatory adjustments of the task set component of interest were possible, to some extent. This work typically revealed involvement of pre-frontal areas when preparing for a task switch (Karayanidis et al., 2003; Nicholson et al., 2005; Karayanidis et al., 2009).

^2^As shown by Shedden and Nordgaard (2001; Töllner et al., 2012a), the PCN is independent of both the amplitude and latency of the non-lateralized N2. Thus, to avoid potential confusion associated with the term “N2pc,” we prefer the neutral term “PCN.”

^3^Analyses of PCN and SPCN amplitudes revealed a significant target position effect for the PCN component (3.50 and 3.25 μV for left and right targets, respectively, *F*_(1,13)_ = 4.81, *p* < 0.05, η^2^_*p*_ = 0.27), and no difference between left and right targets in the SPCN component (*F* = 1.62, *p* = 0.22). Most importantly, target position did not interact significantly with electrode site (ipsi- vs. contralateral), nor with any other experimental manipulation (all *F*s < 2.16, all *p* > 0.16)—indicating that the PCN and SPCN components were not specific for one visual hemifield.
